# Black Mulberry (*Morus nigra* L.): A Review of Attributes as an Anticancer Agent to Encourage Pharmaceutical Development

**DOI:** 10.1155/2024/3784092

**Published:** 2024-11-04

**Authors:** Ana Paula Costa Rodrigues Ferraz, Patrícia de Oliveira Figueiredo, Nídia Cristiane Yoshida

**Affiliations:** Federal University of Mato Grosso do Sul (UFMS), Institute of Chemistry, INQUI, Campo Grande 79074-460/549, Brazil

**Keywords:** apoptosis, cancer, drug, polyphenols

## Abstract

Recent considerations of natural sources as potential anticancer agents have arisen due to the origins of numerous drugs commonly used in chemotherapy. Plant-based drugs, in particular, have attracted attention for offering the advantage of low adverse effects. Among these, the black mulberry plant (*Morus nigra* L.) stands out as a natural source of polyphenols, widely used to treat metabolic dysfunctions and confer benefits on human health. This study explores the potential of this plant as an anticancer agent, examining its effectiveness based on the type of application of the plant extracts or isolated substances, extraction methods, and its potential biological effects on cancer cells. Consequently, this study contributes to a better understanding of the distribution of phytochemicals in *M. nigra* and their applications in the context of cancer field. Among the compounds found in black mulberry are flavonoids, chlorogenic acid, cryptochlorogenic acid, and protocatechuic acid, along with cyanidin-3-*O*-glucoside as the main anthocyanin on the fruit. The phytochemicals derived from *M. nigra* exhibit antinociceptive and antimicrobial activities, while also showing protective effects, such as antioxidant properties that underline their potential as anticancer agents. The black mulberry's roots, stem bark, pulp, and leaves are particularly rich sources of anti-inflammatory compounds. Ethanol and methanol extraction methods appear to be the most effective in cancer management, offering compounds that facilitate the integration of apoptosis induction, cell growth inhibition, and cytotoxicity modulation. These results collectively represent the salient biological attributes that positioned black mulberry as a promising anticancer agent. Therefore, these findings highlight the multifaceted potential of *M. nigra* as an anticancer agent, making a compelling case for further research to advance prospects in the medical field.

## 1. Introduction

Anticancer or antineoplastic drugs are known for their effectiveness in treating cancer malignancy [1]. However, many adverse effects on patients are reported with the use of conventional medicine [2]. In this context, researching natural products for their potential in cancer treatment could make a substantial contribution by delivering the desired anticancer effects with fewer side effects. In the field of cancer research, advanced studies encourage the discovery of natural compounds with anticancer activity, considering that many conventional medicines originated from botanical sources [3].

Numerous plants subjected to *in vitro* research have been identified for their anticancer properties. The majority of these plants exhibit the ability to inhibit malignant cells by preventing DNA damage and by activating apoptosis through secondary metabolites, highlighting their potential role in phytotherapy [4].

Promising new agents for clinical applications are based on selective activities carried out by plants or isolated substances that mediate inhibition of tumor cell proliferation while exhibiting low toxicity, apoptotic mechanisms, and other multifaceted modes of action [5, 6].

Apoptosis stands out as a promising target in the management of anticancer strategies, given that the deregulation of apoptosis intrinsic pathways contributes to enhanced tumor survival [7]. Another noteworthy aspect is inflammation, as its connection with various cancer conditions underscores its significance. Inflammation serves as an organism's response to address infection, tissue injury, or cellular stress, aiming to restore tissue function through repair mechanisms. However, this intricate process involves extensive communication among various immune and nonimmune cells and, unlike normal tissue, cancer induces persistent inflammation, observed as a hallmark in almost every cancer type. The crosstalk between malignant and nonmalignant cells, mediated by factors such as cytokines and chemokines, collaborates with genetic alterations, leading to tumor progression and metastasis. Mechanistically, tumor-associated inflammation produces mutagenic factors, supports angiogenesis, and suppresses antitumor immune responses, which are the crucial steps in facilitating cancer dissemination and metastatic progression [8]. Consequently, the exploration of novel molecules with potential anti-inflammatory targets becomes crucial for the development of innovative pharmaceutical products.

The fruits of the mulberry tree (*Morus* sp.) are consumed all over the world and are considered a rich nutritional source of vitamin C, dietary fiber, malic acid, linoleic acid, proteins, and minerals such as calcium, copper, iron, zinc, selenium, and magnesium. *Morus* species have been reported to be rich sources of secondary metabolites such as phenolic compounds, flavonoids, and anthocyanins [9]. The botanical parts (leaves, fruits, root bark, and branches) of the *Morus* tree are all considered important for nutraceutical purposes, and the compound morusin, isolated from the root bark of *M. alba* [10], is the most promising substance with metabolic functions. The nutrient content of *Morus* sp. fruits depends on the species, soil composition, environmental conditions, and fruit maturity [11]. Based on pH, total soluble solids, and dry weight, *M. alba* fruits are generally recommended for processing, in contrast to *M. nigra*, which is recommended for fresh fruit production [12]. In addition, black fruits have a better flavor due to their low pH value compared to white fruits [12]. Extracting natural compounds from fruit byproducts is also recognized as an advantageous approach, once these materials are considered a rich source of nutrients due to the fact that various parts of the plant, such as residual pulp components (peels, flesh, skin, seeds, and stems), can harbor a diverse range of other valuable nutrients that are an interest to the pharmaceutical industry [13]. Polyphenols are a class of secondary metabolites commonly found on fruits, such as *Morus nigra* L., and these compounds are present in various food sources. However, their bioavailability seems to differ between the various types of polyphenol molecules [14] and, furthermore, the type of extraction also influences the plant's potential based on the interesting molecules presented in the composition of an extract [15]. To date, a comprehensive analysis of the chemical composition and sources of specific characteristics as an anticancer agent that could increase the potential of black mulberry (*Morus nigra* L.) has not been presented so far. This information has provided new perspectives on how black mulberry can be integrated as a source of natural anticancer products.

This review was carried out aiming at summarizing the application of *Morus nigra* L. against various types of cancer, including its greater or lesser potential to be an anticancer agent in relation to the type of extraction and its biological effect on cancer cells.

## 2. Sources and Methodology

### 2.1. Study Design

All methodology was structured according to Synder [16]. This study adopts a systematic review approach to comprehensively assess the evidence of the anticancer effects associated with black mulberry (*Morus nigra* L.) fruits. Data collection involved three independent investigations utilizing the SciFinder, Tropicos, and National Library of Medicine (PubMed) electronic databases. The research specifically delves into *in vitro* and *in vivo* studies exploring Black Mulberry's potential as an anticancer agent, emphasizing molecular anticancer mechanisms. The analysis covers botanical features, the phytochemical profile with anticancer potential, and its modulating effects on metabolism. In addition, extraction methods derived from *M. nigra* and their impact on enhancing or diminishing its potential as an anticancer agent are discussed.

### 2.2. Conduct

Three independent investigations were performed: (1) National Library of Medicine (PubMed) was used for research regarding the following descriptors: “*Morus nigra* L AND anticancer effect” and “Black Mulberry AND anticancer” resulting in 13 article elected, since these articles must present the anticancer subject and full-text format; (2) SciFinder was recruited with three independent investigations: (a) research tasks using “*Morus nigra* L. as Review,” resulted in 30 research articles, from which, one article was elected with anticancer effect versus black mulberry and 29 articles were excluded for the not anticancer theme; (b) research tasks using “*Morus nigra* L. as Antitumor,” resulted in 30 results filtered, from which, 12 articles were recruited as antitumor potential, 8 articles were excluded for not targeting anticancer outcomes, one was excluded for not full-text, one was excluded for not being a research article, and 9 articles were excluded since they were duplicated; and (c) research tasks using “*Morus nigra* L. as Anticancer,” resulted in 28 results, from which, 22 were excluded for duplicates and 6 were excluded for no target anticancer outcomes; (3) Tropicos platform (Tropicos.org. Missouri Botanical Garden) using “*Morus*” as a descriptor, resulted in 141 items related to *Morus* specimen and for “*Morus nigra* L.,” from which, 141 were excluded for not theme and one article about *Morus nigra's* taxonomy was recorded. Records identified for inclusion as a systematic review were 17 articles.

### 2.3. Data Abstraction and Analysis

Elected boolean descriptors used in this study offered keys to investigating black mulberry as an anticancer agent, offering the precision of analysis on the route: “*Morus nigra* L AND anticancer effect,” “Black Mulberry AND anticancer,” “*Morus nigra* L. as Review,” “*Morus nigra* L. as Antitumor,” “*Morus nigra* L as Anticancer,” “*Morus,*” and ”*Morus nigra* L.” 241 studies were located in the PubMed, SciFinder, and Tropicos. The data abstraction was performed since the elected studies were counted even from the article numbers regarding the amount of excluded or included studies, integrating the systematic review.

**Figure 1:**
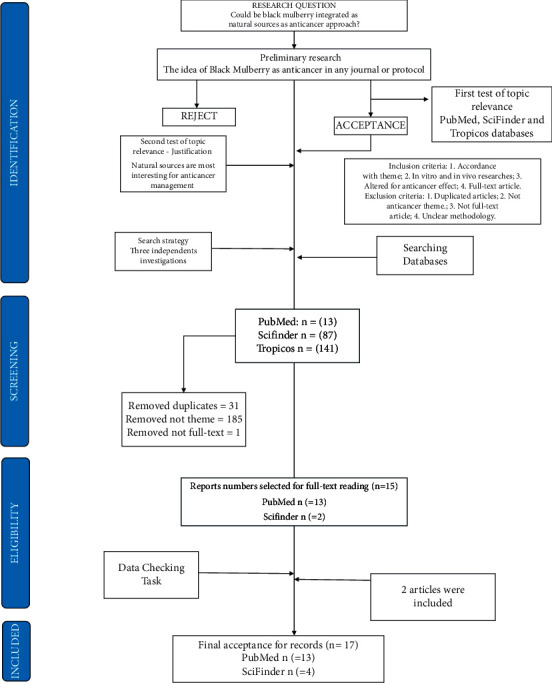
Study data abstraction and analysis, culminated in a total count of 17 elected studies.

For this selection, these studies were filtered by an inclusion or exclusion criteria, which contemplate for the first inclusion criteria: in accordance with the anticancer theme. The anticancer subject was indicated as a major inclusion criterion since this study has a purpose of investigating the several possibilities that can enhance black mulberry as an anticancer agent. For this, the PubMed plus SciFinder were delegated with anticancer and antitumoral tasks and Tropicos was elected as the botanic data finder, which looks at the diversity regarding the *Morus* genus, specimens, and so on. Following these inclusion criteria, the elected articles should be *in vitro* or *in vivo* methodologies; also, they have been shown as full-text articles for revision purposes. Exclusion criteria were considered, which were studies' duplicates, following not on anticancer subject, or no clearer methodology. Filtered by anticancer theme and other criteria for selection (see Figure 1), the number dropped to 17 studies. Figure 1 exposed the data abstraction and analysis for this study.

## 3. Results and Discussion

### 3.1. Botanical, Chemical, and Phytochemical Characteristics of *Morus nigra* L

*Morus* species are widely known as an economical, fast-growing deciduous tree native to Asia, North America, Europe, and Africa, widely cultivated for silk production [11].

The *Morus* genus comprises approximately 24 species, with 11 principal species distributed worldwide, in addition to one subspecies and over 100 varieties [12]. *M. alba* (white mulberry), *M. nigra* (black mulberry), and *M. rubra* (red mulberry) are the most common species [9]. The botanical characteristics of *M. nigra* are summarized (see Table 1) and illustrated (Appendix 1).

The main nutritional components found in *M. nigra* are 82.40% of moisture, 0.9% of protein, 13.8% of carbohydrates, 11.7% of fiber, and 0.5% of fat. Other components are potassium (1270 mg/100 g), total phenols (880 mg/100 g FW), alkaloids (630 mg/100 g FW), calcium (470 mg/100 g), sodium (272 mg/100 g), magnesium (240 mg/100 g), iron (77.6 mg/100 g), and ascorbic acid (15.37 mg/100 g FW) [12, 18]. At the same time, when comparing mulberry fruits with *M. alba* and *M. mongolia*, *M. nigra* is the richest in anthocyanins (main pigments: cyanidin-3-glucoside and cyanidin-3-glucosyl rhamnoside) [13] and flavonol content [19], being considered a promising source of these compounds among mulberry species. These phytochemical compounds present relevant beneficial effects on human health through a variety of plant substances, generally with different structures capable of health-promoting effects. Cyanidin-3-glucoside stands out as the predominant anthocyanin present in natural plants. This particular anthocyanin variant gives rise to phenolic acids and conjugates, such as phenylacetic, hippuric, and phenylpropanoic acids, through the process of human digestion [14]. Notably, a heightened consumption of fruits and vegetables is recognized for its protective effects and its association with a reduced risk of various types of cancer, cognitive impairments, diabetes, and other relevant diseases [15].

Wang et al. [20] analyzed 13 cultivars of black mulberry fruit extracted in a mixture of methanol/water (80:20, v/v) acidified with hydrochloric acid (0.5%), aiming at obtaining hydroalcoholic extracts. The chemical analysis allowed the identification of 55 types of phenolic compounds, including chlorogenic, cryptochlorogenic, and protocatechuic acids as the main phenolic compound contents, even assessed in different cultivars of *M. nigra* [20]. In addition, Table 2 contains a description of the main phytochemicals found in black mulberry.

**Figure 2:**
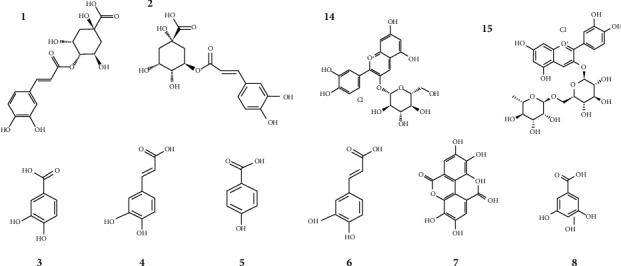
Relevant phytochemical substances of *Morus nigra* L., presented in order of highest to lowest content in the plant (from left to right side).

The major phytochemicals, in order of relevance, are shown in this research (see Figure 2).

Pharmacological properties of black mulberry: The principles of nutraceutical potential for basic characteristics related to cancer development.

**Figure 3:**
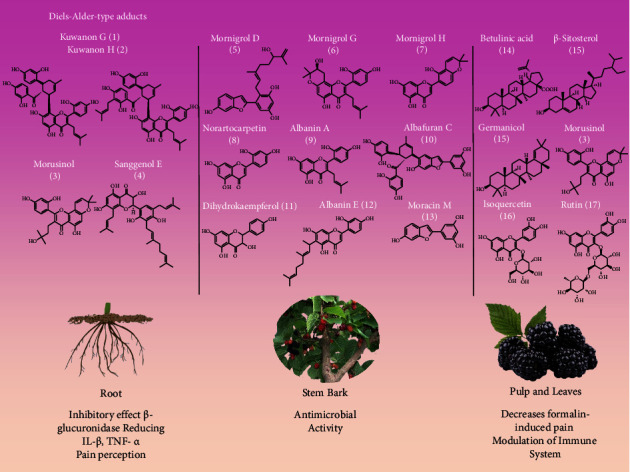
Phytochemicals of *Morus nigra* L. with various effects. Root: presence of Diels–Alder type adducts (kuwanon G (1) and kuwanon H (2); morusinol (3); sanggenon E (4); stem bark: mornigrol D (5); mornigrol G (6); mornigrol H (7); norartocarpetin (8); albanin A(9); albafuran C (10); dihydrokaempferol (11); albanin E (12); moracin M (13); and pulp and leaves: betulinic acid (14); *β*-sitosterol (15); germanicol (16); morusinol (3); isoquercetin (16); and rutin (17)).

The *M. nigra* plant produces a typical Cerrado fruit [24], the black mulberry that has been used as a promising nutraceutical resource with antinociceptive, anti-inflammatory, antimicrobial, antidiabetic, antiobesity, antihyperlipidemic, antiatherosclerotic, and protective activities, such as antioxidant, neuroprotective, hepatoprotective, renal, and gastroprotective [18]. The nature of the plant is a plethora of phytocomplexes that may be involved in multiple actions against the factors that cause cancer [25]. The main phytochemicals responsible for possible anticancer characteristics and the main factors in the development of cancer were related (see Figure 3 and Table 3). In relation to its flavonoid content, the main phenyl flavonoid in blackberry is morusin, which showed inhibitory action against the abdominal constriction responses induced by acetic acid, potentiating the anti-inflammatory cytokine IL-10 and other pathways such as nitric oxide (NO) and nuclear factor kappa-light-chain-enhancer of activated B cells (NF-*κ*B). In addition, morusin decreased the secondary phase of formalin-induced pain, known as the anti-inflammatory circuit, which includes these pathways in the modulation of the antinociceptive effect [18]. From the stem bark of *M. nigra*, some compounds (5. mornigrol D, 6. mornigrol G and 7. mornigrol H, 8. norartocarpetin, 11. dihydrokaempferol, 9. albanin A, 12. albanin E, 13. moracin M and 10. albafuran C, see Figure 3) were isolated during a study using an anti-inflammatory search approach. *M. nigra* can decrease the levels of proinflammatory cytokines such as IL-1*β*, TNF-*α*, NO, and IFN-*γ* [18]. Hooshmand et al. [35] prepared a mixture (1:1, w/v) of *M. nigra* fruits and 50% aqueous ethanol (acidified with 1% HCl) crushed by a juicer to obtain an extract with anthocyanins. This acidified aqueous ethanolic extract showed a hepatoprotective effect, reducing the pathways of cancer formation [35]. Almutairi et al. [36] demonstrated that using dried leaves of *M. nigra* has a high inhibition percentage of albumin denaturation and antioxidant potential regarding the anticancer context [36]. Leaf and fruits showed interesting pharmacological properties as antimicrobials using ethanol/methanol–based extracts [18]. Souza et al. [37] demonstrated a strong antibacterial activity from dried and pulverized leaves extract prepared with 95% ethanol (w/v) [37]. Hago et al. [38] showed that ethanolic extracts from leaves of *M. nigra* possess promising antidiabetic (hypoglycemic) effects [38]. Black mulberry has been identified as having potential antiobesity properties, as indicated by a study conducted by Fan et al. [39] in 2020. In this study, 48 fattened pigs were supplemented with 5% (w/w) mulberry leaf powder. The researchers observed a reduction in fat mass, attributing it in part to the increased lipolysis in the pig model. These findings suggest that black mulberry could provide a foundation for the development of antiobesity drugs [39]. Other study also explored repercussions on the lipidomic system and highlighted the antimelanogenic properties [18]. The use of leaf extract, with ethanol as the extracting solvent, has demonstrated potential in the management of obesity. Physio's pathways that are involved in inflammation settings can be explored indirectly such as in the context of obesity, in which mulberry is a potential protective agent. Extracts enriched with various phenolic compounds and isoprenylated flavonoids from *M. nigra* demonstrate an ability to enhance the activation of peroxisome proliferator-activated receptor gamma (PPAR*γ*) also involved in DNA level on cell survival and lipid metabolism [40]. However, in the realm of obesity and metabolic disorders, particularly noteworthy is the inhibitory effect on metalloproteinases (MMPs), as highlighted in *in vivo* research studies [18]. These findings underscore the potential of black mulberry extracts in addressing obesity-related pathways, with specific attention to MMP inhibition. The antimelanogenic property is described as extracts obtained from the stem and roots of *M. nigra* [18]. In spite of that, *M. nigra* leaves extracted with aqueous ethanol 95% showed the presence of chlorogenic acid, rutin, and isoquercetin as major compounds. De Freitas et al. [41] found the black mulberry leaf extract as a promising natural source against hyperpigmentation, which commonly affects the skin [41].

### 3.2. *Morus nigra* and Its Anticancer Applicability

Careful evaluation and the judicious selection of solvents, dosage, and plant extraction methods are recognized as fundamental criteria for pharmacological applications in human health [42, 43]. Extracts utilizing ethanol and methanol as solvents have been identified as having the most pronounced beneficial effects as carriers of phenolic compounds [44, 45]. The selection of the solvent, along with its concentration, plays a crucial role in determining the efficacy of these extracts [45].

In the context of colon anticancer activities, lyophilized black mulberry extracts prepared with water have been investigated. Fruit extracts were obtained using a 75% (v/v) ethanol/water solvent through both heated and unheated methods. Notably, both heated water and ethanol extracts demonstrated greater efficacy compared to their unheated counterparts. These extracts exhibited significant effects in inhibiting cell growth, altering cell morphology, elevating intracellular Ca^2+^, reducing mitochondrial potential, and increasing the production of reactive oxygen species (ROS) [46]. The modulation of ROS content in the cancer environment is recognized as a compelling area for controlling the balance between preventing and inducing cell death. Augmenting antioxidant levels has the potential to trigger apoptosis or autophagy in cancer cells [47]. *M. nigra* is commonly acknowledged as a source of antioxidants, thanks to its rich polyphenol and anthocyanin profiles [13, 48, 49]. This establishes *M. nigra* as a promising candidate for interventions targeting ROS modulation and the associated mechanisms in cancer therapy.

Qadir et al. [50] demonstrated a dose-dependent inhibition of the human colorectal adenocarcinoma cell line with epithelial morphology (HT-29) using aqueous methanol and *n*-hexane extracts from *M. nigra* leaves [50]. Subsequently, De Freitas [41] investigated tyrosinase inhibition, a crucial pathway in the cosmetics industry. Deregulated melanogenesis was effectively mitigated by a standardized extract of *M. nigra* leaves, rich in chlorogenic acid, rutin, and isoquercetin. This extract serves as a novel source of tyrosinase inhibitors, offering the potential to prevent hyperpigmentation [41]. In another study, the powdered leaf sample was dissolved in 100 mL of ethanol, methanol, and water, and the extracts were tested at concentrations between 0 and 3200 *μ*g/mL against HT-29 cancer cells. Promising effects on apoptosis of MV1A and HT-29 cells were observed, particularly with the aqueous extract at a dosage of 160 *μ*g/mL [51]. Ahmed et al. [52] extracted fresh and dried fruits with 70% ethanol. The ethanolic extract showed inhibition of human breast cancer cells' (MCF-7) growth, formation of apoptotic bodies, reduction of nuclear abnormalities, and fragmented DNA. This effect was attributed to natural polyphenols with chemopreventive and/or chemotherapeutic capacity, including anthocyanin, which acts as an anticarcinogen against various types of cancer [52]. In a previous study, Çakıroğlu et al. [53] analyzed *M. nigra* fruits homogenized with 200 mL of 80% (v/v) methanol to obtain a methanolic extract. This extract was tested as a drug candidate against HT-29 cells, along with other substances such as morniga-G (MorG), a leptin from *M. nigra* [53]. The results pointed out promising inhibition properties of HT-29 cells, particularly the Mor G.

Corroborating the potential of blackberry, other species of the *Morus* genus have been shown to have anticancer potential. Hamdan et al. demonstrated anticancer properties against hepatocellular carcinoma (HepG2), mama (MCF-7), and cervical carcinoma (HeLa) cell lines, which were treated with dried and powdered stems, leaves, and fruits from *M. macroura*, known as white mulberry, macerated with 80% aqueous ethanol at room temperature. In addition, fractions partitioned into ethyl acetate from these parts of the plant's organs were analyzed by high-performance liquid chromatography-electrospray ionization tandem mass spectrometry (HPLC-ESI-MS) and also exhibited robust cytotoxic activity [54]. Tumor cell destruction, commonly referred to as cytotoxicity, constitutes a fundamental aspect in the development of chemotherapeutic drugs, occupying a significant 41.9% of the research area in oncology. This prominence arises from the fact that the therapeutic efficacy of the majority of anticancer drugs employed in chemotherapy is rooted in their ability to induce toxicity in living cancer cells. These drugs disrupt various crucial aspects such as tumor-specific cell signaling pathways, metabolism (including glucose utilization and blood supply), genetics, drug resistance mechanisms, and cell proliferation [55]. In testing against the breast adenocarcinoma cell line (MDA-MB-231), heightened cytotoxic effects were observed at a 10% concentration of fresh *M. nigra* fruits [56]. The recent trend in developing cytotoxic drugs aims to destroy cancer cells circulating in the body with minimal side effects, providing a less toxic treatment for healthy cells [55].

In another study, blackberry leaves, *Urtica urens* L., and *Glycyrrhiza glabra* L. were macerated with 500 mL of ethanol at 25°C–30°C. The plant extracts showed inhibitory activity against HepG2 cells and mouse L cells transfected with the gene coding for the human cellular receptor for poliovirus (L20B) [57]. Among the active plant compounds, morusin induces autophagy and apoptosis, associated with an autophagic inhibitor, taken together, increasing the efficacy of morusin attributed as a combined treatment [58].

**Figure 4:**
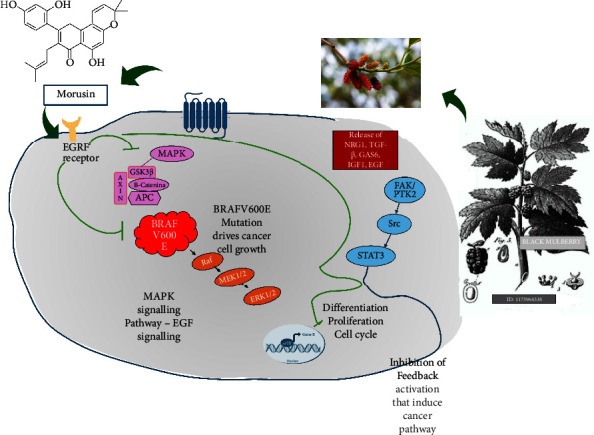
Morusin as a natural anticancer agent and its mechanism for inhibiting BRAF-mutant melanoma and MAPK pathway and suppressing the feedback activation that activates the STAT3 pathway [59]. This inhibitory effect was discovered through the inhibition of the feedback activation of the STAT3/SOX2 pathway indirectly induced by differentiation/proliferation/cell cycle from BRAFV600E mutation. These findings underscore the critical role of these pathways in potentially reversing drug resistance in melanoma.

Apoptosis, another target of new and promising anticancer therapies, through the activation of pathways, causes the death of cells. Mandatory effects from morusin as an anticancer agent were expressed (see Figure 4).

Turan et al. [60] also revealed apoptotic and antiproliferative effects from black mulberry mature fruits extracted with DMSO. The extract of *M. nigra* fruits revealed apoptotic and antiproliferative effects on human prostate adenocarcinoma (PC3) cells [60]. In addition, Erden [61] found similar outcomes for fresh black mulberry fruits extracted in 80% ethanol at a ratio of 1:20 (w/v) for 12 h. At a concentration of 75 *μ*g/mL, this extract acted as a cell death inducer against HT-29 cells, leading to increased DNA damage and reduced cell viability within 24 h. This effect was associated with elevated Bax/Bcl-2 levels and decreased p53 and procaspase-3 levels [61]. The comprehensive summary of studies investigating the potential anticancer activity of *M. nigra* is provided in Table 4 (see Table 4).

## 4. Conclusions

Botanical features were meticulously examined in conjunction with substances harboring anticancer potential in this study. Notably, flavonoids, along with organic acids and chlorogenic, cryptochlorogenic, and protocatechuic acids, are major phenolic compounds found, alongside cyanidin-3-*O*-glucoside as a major anthocyanin find in black mulberry fruits. Phytochemicals of *Morus nigra* L. revealed antinociceptive, antimicrobial, and antioxidant properties. Bioactive compounds such as kuwanon G-H, morusinol, and sanggenon E were identified in the roots, while steam bark contained mornigrol D, mornigrol G, mornigrol H, norartocarpetin, albanin A, albafuran C, dihydrokaempferol, albanin E, and moracin M. Betulinic acid, *β*-sitosterol, germanicol, morusinol, 3-*O*-glycoside/isoquercetin, and quercetin 3-*O-*rutinoside/rutin are compounds found in pulp and leaves of black mulberry, which were pointed out for being responsible for the anti-inflammatory effect, inhibition of release of β-glucuronidase reducing side effects on chemotherapy, action in formalin-induced pain models and antimicrobial effect.

The integration of apoptosis induction, reversal of drug resistance in melanoma pathways, inhibition of cell growth, and low cytotoxicity toward normal cells, coupled with enhanced cell survival, are the key attributes that make *Morus nigra* L. as a highly promising potential anticancer agent. Apoptosis induction, toxicity modulation, inflammation control, and cell proliferation regulation are recognized as fundamental factors in establishing robust *in vitro* research platforms for potential *in vivo* and clinical applications. *Morus nigra* L. has shown satisfactory performance in these aspects. Consequently, further explorations into the potential of *M. nigra* as an anticancer agent could yield substantial benefits for the field of oncology research.

## Figures and Tables

**Table 1:** Botanical description of *Morus nigra* L.

Kingdom	Plantae
Phylum	Tracheophyta
Class	Magnoliopsida
Order	Rosales
Family	Moraceae
Genus	*Morus* L.
Specimen	*Morus nigra* L.
Usual name	*Black mulberry, wild blackberry, blackberry*

*Note: Source:* Integrated taxonomic information system–*Morus nigra* L. (1753). In: Sp. Pl.: 986. [17].

**Table 2:** Main phytochemicals present in the fruit of black mulberry samples (*Morus nigra* L.).

**No.**	**Phytochemicals**	**Methods**	**Average content (mg/kg DW)**	**Reference**
	**Phenolic compounds∗**			

1.	Cryptochlorogenic acid	UPLC-ESI-MS/MS	81.50–422.18	[20]
2.	Chlorogenic acid	Folin–Ciocalteu	56.94–306.01	[20]
3.	Protocatechuic acid		14.32–101.74	[20]
4.	Caffeic acid		0.66–6.52	[20]
5.	*p*-Hydroxybenzoic acid		0.53–4.71	[20]
6.	4-Hydroxycinnamic acid		0.40–3.28	[20]
7.	Ellagic acid		1.09–2.95	[20]
8.	Gallic acid		0.06–1.68	[20]
9.	Vanillic acid		0.14–1.02	[20]
10.	Salicylic acid		0.07–0.84	[20]
11.	Ferulic acid		0.09–0.52	[20]
12.	Syringic acid		0.01–0.35	[20]
13.	Total phenolic contents (mg/g DW)∗		161.26–851.9	[20]

	**Anthocyanins**		**(mg/g)**	

14.	Cyanidin-3-*O*-glucoside	UPLC-TUV/QDa	8.2168	[3, 21]
15.	Cyanidin-3-*O*-rutinoside		2.8578	[3, 21]
16.	Pelargonidin-3-*O*-glucoside		0.2539	[3, 21]
17.	Total anthocyanins (mg/g)	Folin–Ciocalteu/HPLC	1.88–0.01	[3, 22]

	**Flavonoids**		**(mg/g)**	

18.	Isoquercetin		0.1639	[3, 21]
19.	Rutin	UPLC-TUV/QDa	0.4498	[3, 21]
20.	Quercetin		0.0716	[3, 21]
21.	Total flavonoids (mg GAE/100 g)	Aluminum chloride colorimetric method	276	[3, 23]

^∗^The content variation among 13 different cultivars of *Morus nigra*. DW, dry weight.

**Table 3:** Phytochemicals obtained from *M. nigra* related to anticancer potential.

No.	Therapeutic properties	Phytochemical compounds	Plant material/study design	Therapeutic perspective	Reference
1.	Antinociceptive	Morusin (prenylflavonoid)	Root bark/mice	Inhibitory effect/23.8–142.9 *μ*mol/kg	[26]
		Morusin (prenylflavonoid)	Root bark/*in vivo*	Pain perception/ID_50_ value of 72.1 mmol/kg	[27]
		Cyanidin-3-*O*-glucoside, rutin, and isoquercetin used as mixture	Fruits/RAW 264.7 cells	Dose-dependent decreases formalin-induced pain (based on levels, cytokines were significantly inhibited or scavenged by total compounds previously tested with this effect performed concerning the 50 e 100 mg/kg in mice1)	[21]

2.	Anti-inflammatory	Betulinic acid, *β*-sitosterol, and germanicol	Leaves/rats	Inhibited the formation of granulomatous tissue/100–300 mg/kg/HPLC fingerprint/model of chronic inflammation using cotton pellet-induced fibrovascular tissue growth in the rats-100 and 300 mg/kg of the extract produced 32.9% and 49.5%, respectively, and significantly inhibited the weight of granulomatous tissues (IC_50_ of the extract: 71.1 mg/kg) Inhibitory values of edema 3 h postcarrageenan were 32%, 50.0%, and 53.1% for 30, 100, and 300 mg/kg of the extract (IC_50_ of the extract: 15.2 mg/kg)	[28]
		Mornigrol D, G, and H; norartocarpetin	Barks/rats	Inhibition of release of b-glucuronidase/10-5 mol/L (percentage of inhibition of malondialdehyde formation (532 nm))	[29]
		Morusinol (IC_50_ = 4.3 ± 0.09 *μ*M), Diels–Alder adducts, soroceal, and sanggenon E isolated with 2′,4′ oxidation pattern (IC_50_ = 4.0 ± 0.12 *μ*M)	Root Extract/THP–1 human monocytic leukemia cell line	1 *μ*M of total compounds Diminish IL-1*β* (ELISA kit)	[30]
		Sanggenon E (IC_50_ = 4.0 ± 0.12 *μ*M) and mulberrofuran Y (IC_50_ = 4.8 ± 0.19 *μ*M)	Root extract/THP–1 human monocytic leukemia cell line	1 *μ*M of total compounds reducing the secretion of TNF- *α* [3]. (ELISA kit)	
		Morusinol (IC_50_ = 4.3 ± 0.09 *μ*M) and mulberrofuran H (IC_50_ = 3.2 ± 0.13 *μ*M)	Root extract/THP–1 human monocytic leukemia cell line	Effect nearly twice that of prednisone/most effective (*p* > 0.001) with activity more potent than prednisone	[30]
		Extract rich in quercetin 3-O-rutinoside/rutin and quercetin 3-O-glycoside/isoquercetin	Pulp and leaf extracts/C57BL/6 mice	Decreased leukocytes in bronchoalveolar lavage fluid and serum levels of TNF in septic animals with 100 µL at a dose of 500 mg.kg of leaf and pulp extract (reduced significantly by ANOVA–one way followed by Bonferroni's posttest	[31]

3.	Antioxidant	Geranyl flavonoids (5′-geranyl-5,7,2′,4′-tetrahydroxyflavone kuwanon E and kuwanon U), chalcones (2,4,2′,4′- tetrahydroxychalcone morachalcone A), arylbenzofurans (macrourin B, moracin O, moracinoside M, morunigrol C, and morunigrol D), coumarins (mulberroside D, xerobside 5,7- dihydroxycoumarin-7-O-*β*-D-glucopyranoside 7-[[6-O-deoxy-*α*-L-mannopyranosyl]-[*β*-D-glucopyranosyl]oxy]-2H-1-benzopyran −2 -one 5,7-dihydroxycoumarin-7-[6-O-*β*-D-apiofuranosyl-*β*-D- glucopyranoside). Abundant in anthocyanin	Total/plant/Review	Active against oxidative stress in various clinical conditions∗	[32]
		Total flavonoids	Total plant/*in vitro/in vivo*	*In vitro*, the clearance rate of hydroxyl radicals and superoxide radical anion increased the concentration of the total flavonoids (0–1.05 mg/mL). *In vivo:* MDA in serum and liver decreased and increased SOD, CAT, and GSH-PX in blood and liver also Langerhans cells in the spleen (different dosages)∗	[19]

4.	Antimicrobial	Total flavonoid extract (TF)	Fruits/*in vitro*	TF (1.8 mg/mL) inflammatory pain caused by bacteria (*E. coli, P. aeruginosa, and S. aureus*)∗	[21]
		Phytocosmetic formulation from leaves with rutin and isoquercetin as major components	Leaves/in vitro	25 mg/mL of stock solution and 20% emulsion with better results against strains of *Staphylococcus aureus*, methicillin-resistant *Staphylococcus aureus* (MRSA), and *Salmonella choleraesuis*∗	[33]
		Stilbenoid oxyresveratrol 1, a 2-arylbenzofuran moracin M2, cyclomorusin 3, morusin 4, kuwanon C5, and a derivative of kuwanon C6	Steam bark and wood/in vitro	Serial microdilution method; activities against *Staphylococcus aureus, Bacillus subtilis, Micrococcus flavus, Streptococcus faecalis, Salmonella abony,* and *Pseudomonas aeruginosa*∗	[34]

^1^H. Chen, W. J. Pu, D. Liu et al., “Anti-inflammatory and Antinociceptive Properties of Flavonoids from the Fruits of Black Mulberry (*Morus nigra* L.)” PLoS One, vol. 11, no. 4, pp e0153080., PMID: 27046026.

^∗^These results were not applicable for justifies concerning activity methodology, in fact, were literature reviews and antibacterial activities with minimum bacterial concentrations (MBC) tests.

**Table 4:** Insights from research studies exploring the anticancer potential of *Morus nigra* L.

No.	Parts of the *M. nigra* plant	Samples' extraction	Dosage/tissue target/experimental design	Therapeutic potential	Ref.
1	Leaves	300 g/4.000 mL of *n*-hexane and 70% aqueous methanol, 7 days	1, 10, 25, 50, and 100 *μ*g/mL/HeLa cell line/*in vitro* study	100 *μ*g/mL inhibited 89.5%–32.0% of the HeLa cell line	[50]
2	Leaves and fruits	Decoction	Unspecified cancer	Unspecified cancer	[62]
3	Leaves	Air-dried powdered in aqueous ethanol 95%, divided into 5 batches of 210 g and extracted with 1050 mL aqueous ethanol 95% at room temperature for 10 days with daily manual agitation	Range from 2000 µg/mL to 0.98 *μ*g/mL/one human keratinocyte cell line (HaCat), one fibroblast cell line (L-929), and one melanoma cell line (B16F10)/*in vitro* study	Development of an herbal medicine/low toxicity to cell lines that constitute skin/Promising topic cosmetic	[41]
4	Sour black mulberry samples	80% ethanol for 12 h	10–100 *μ*g/mL/HT-29 human colon cancer cells/*in vitro* study	Increased DNA damage and reduced cell viability/mediate cell death in caspase-3 by decreasing mutant p53 expression	[61]
5	Black mulberry juice	Full matured stage. Black mulberry fruit juice (BMFJ) concentrations alone (1/1, 1/2, 1/4, 1/8 dilutions)	(1/1, 1/2, 1/4, 1/8) dilutions/human lymphocytes–immune system/*in vitro* study	No genotoxicity/protection of chromosomal damages	[63]
6	Fruits	Fruits were extracted using a blender and diluted 1 mL of *M. nigra* extract into 9 mL of culture medium	Stock solution and serial dilutions (10%, 4%, 2%, 1.33%, and 1%)/breast cancer (MDA-MB-231) and prostate cancer (PC3) cells/*in vitro* study	High cytotoxic effect at a concentration of 10%/especially effect against breast cancer cells	[56]
7	Lyophilized black mulberry	1. 0.12 g/10 mL with water at 20°C for 20 min, water was adjusted for 20 mL and heated/0.12 g in 75% ethanol-water solvent, water was adjusted for 20 mL and heated	Human colon cancer HCT-116 cells/*in vitro* study	Heat treatment increases the anticolon cancer effect through inhibition of cell growth, alters morphology, generates more ROS and Ca^2+^, and reduces mitochondrial potential	[46]
8	Mature fruits of *M. nigra*	DMSO extract	Human prostate cancer cells (PC3)/370 and 666 *μ*g/mL*/in vitro* study	Arrested the cell cycle of PC3 and G1 phase, induced apoptosis via increased caspase and also reduced mitochondrial membrane potential	[60]
9	ANS88 black mulberry genotype	Tap water optimum for rooting of mulberry cutting/lyophilized ethanolic berry extracts	(0.5, 1, 1.5, 2, and 2.5) mg/mL/*in vitro* study	Higher antioxidant capacity and reduced cancer cell viability	[64]
10	Fresh and dry fruit extracts	50 g with 10x (w/v) 70% ethanol	Fresh black mulberry extract - 100 *μ*g/mL/300 *μ*g/mL/dried black mulberry extract-100 *μ*g/mL/300 *μ*g/mL/human breast cancer cell line (MCF-7)/*in vitro* study	Decreased mitotic index records and better antiproliferative effect (decreased in dose and time-dependent manner)	[52]
11	Leaves	Tap water, 100 g in 500 mL of 99% ethanol in 25°C–30°C, 7 days	HepG2 hepatocarcinoma and L20B polioviruses/3.9, 7.81, 15.62, 31.25, 62.5, 125 and 250 mg/mL/*in vitro* study	*Morus nigra* L. showed the lowest cytotoxicity; phenolic compounds may play the inhibitory role against these cell lines	[57]
12	Fruits	50 g/200 mL 80% v/v methanol	Human colorectal adenocarcinoma HT-29/10%, 1%, 0.1% of *M. nigra* extract/*in vitro* study	Reduced cell viability, after 48 h decreased significantly on the cell viability	[53]
13	Fruits	Mixture (1:1w/v) of the fruits and 50% aqueous ethanol acidified with 1% HCL was crushed by a juicer	Hepatocellular carcinoma/male Sprague–Dawley rats/100 mg/kg/day for 4 months/*in vivo* study	Extract based of *M. nigra* significantly increased survival, reduced the size of HCC nodules, improved liver oxidant/antioxidant status, prevented blood changes (bilirubin, AST, ALT, and ALP), decreased the expression of Wnt4 and *β*-catenin/in conclusion, the extract exhibits antiproliferative effect through inhibiting oxidative stress and Wnt4/*β*-catenin and prolongs survival	[35]
14	Leaves–fabrication of zinc oxide nanoparticles from *M. nigra*	Rinsed methodically under tap water and cut into tiny pieces, and added to a circulating oven (50°C) for drying completely ⟶ 2 g of the extract was poached with distilled water for 15 min/synthesis of ZnONPs ⟶ 0.1 M of Zn (NO_3_)_2_·6 H_2_O on 50 mL of ddH_2_O was prepared and affixed to 10 mL of extract into the solution in 80°C to approximately 4 h	AGS gastric cancer cells/the MN-ZnONPs dosage (0–100 *μ*g/mL)	MN-ZnONPs induced apoptosis through arresting the cell cycle and preventing antiapoptotic proteins, by enhanced ROS, revealing improved anticancer activity by decreasing mitochondrial membrane potential (MMP)	[65]
15	Morniga-G (MorG) and Morniga-M (MorM) were purified from the bark of a black mulberry tree	Barkmeal (50 g) was extracted in 500 mL of 0.2 M NaCl containing 0.2% (w/v) ascorbic acid by pH 7 by continuous stirring overnight in the cold (2°C) according to Van Damme et al. [66]	Parental Jurkat A3 leukemia cell line, caspase-9-deficient Jukart cells, caspase 8-and 10-doubly deficient Jukart cells, and peripheral blood mononuclear cells (PBMC-)/Morniga-M and Morniga-G (2.5–25 *μ*g/mL)*/in vitro* study	Morniga-G induces the cell death of Tn-positive leukemic cells via concomitant O-glycosylation-, caspase-, and TRAIL/DR5-dependent pathways	[67]
16	Barks from *M. nigra* provided a series of methylated stilbenes 15–19	*M. nigra* stem barks (4 kg) were exhausted with methanol and evaporated under reduced pressure to yield 570 g and submitted to the isolation process according to Abbas et al. [68]	MCF-7 and HepG2 for antiproliferative activity/*in vitro* study	A new 20, 3, 40-trimethoxy-5-hydroxy-trans-stilbene has remarkable activity against MCF-7 cells with IC_50_ 12.5 *μ*M/kuwanon C (5) showed the highest antiproliferative activity with IC_50_ 3.92 *μ*M against MCF-7 and 9.54 *μ*M against HepG2	[69]
17	Whole fruits of *M. nigra*	Powered air-dried fruits of *M. nigra* (20 kg) were extracted with 95% EtOH (3 × 30 L × 2 h) was prepared to obtain a total extract (7.7 kg)	LO2, SW1990, Aspc-1, Bxpc-3, MM231, MM468, MCF-7, HeLa, Hep3B/methyl caffeate dosage 0.2−200 *μ*M/*in vitro* study	Methyl caffeate exhibited effective inhibition against s 3-phosphoglycerate dehydrogenase (PHGDH) and caused apoptosis of cervical cancer cells in micromolar concentrations	[70]

## Data Availability

The data that support the findings of this study are available from the corresponding author upon reasonable request.

## Supporting Information

Appendix 1: Black mulberry (*Morus nigra*) at various stages of growth and fruit development. (1) Seeding; (2) leaves; (3) *Morus* tree; (4) ripe fruits; (5)–(7) different stages of fruit development (photographs taken by Guilherme Fiorese Silva Moraes, 2024).
