# Supplementary material for: Black Mulberry (Morus nigra L.): A Review of Attributes as an Anticancer Agent to Encourage Pharmaceutical Development
Source: Adv Pharmacol Pharm Sci. 2024 Nov 4;2024:3784092. doi: 10.1155/2024/3784092 (PMC11554416; doi:10.1155/2024/3784092)
Supplement: Supporting Information — Additional supporting information can be found online in the Supporting Information section. [file 3784092.f1.docx]

**Supplementary Materials**

Appendix 1 – Photographic montage of the black mulberry (*Morus nigra*) at various stages of growth and fruit development. 1) Seeding; 2) Leaves; 3) Morus tree; 4) Ripe fruits; 5-7) different stages of fruit development (Images were provided by the authors, with photographs taken by Guilherme Fiorese Silva Moraes, 2024).

**
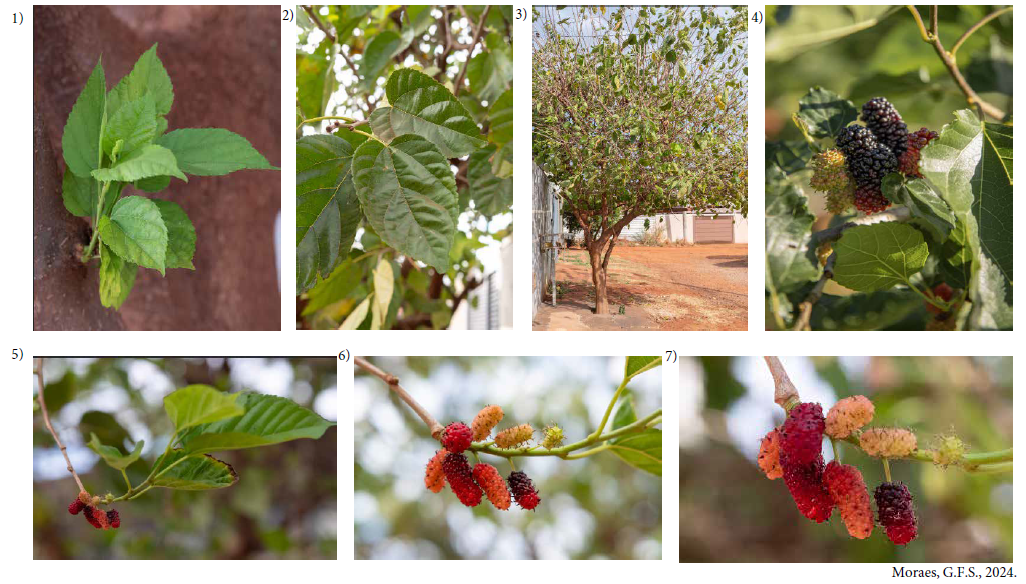
**
